# Case Reports: Why Bother?

**DOI:** 10.1016/j.jaccas.2024.102541

**Published:** 2024-09-18

**Authors:** Mirvat Alasnag

**Figure:**
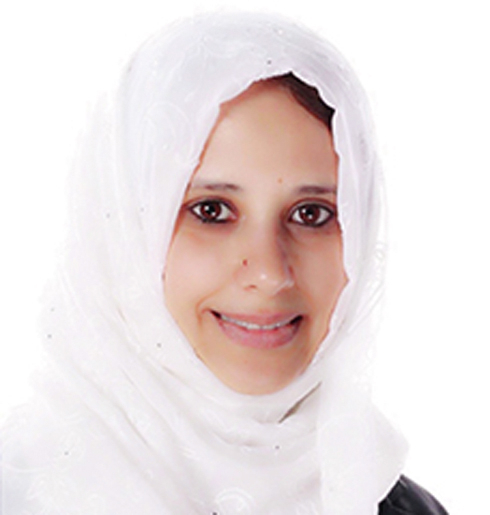

**Figure:**
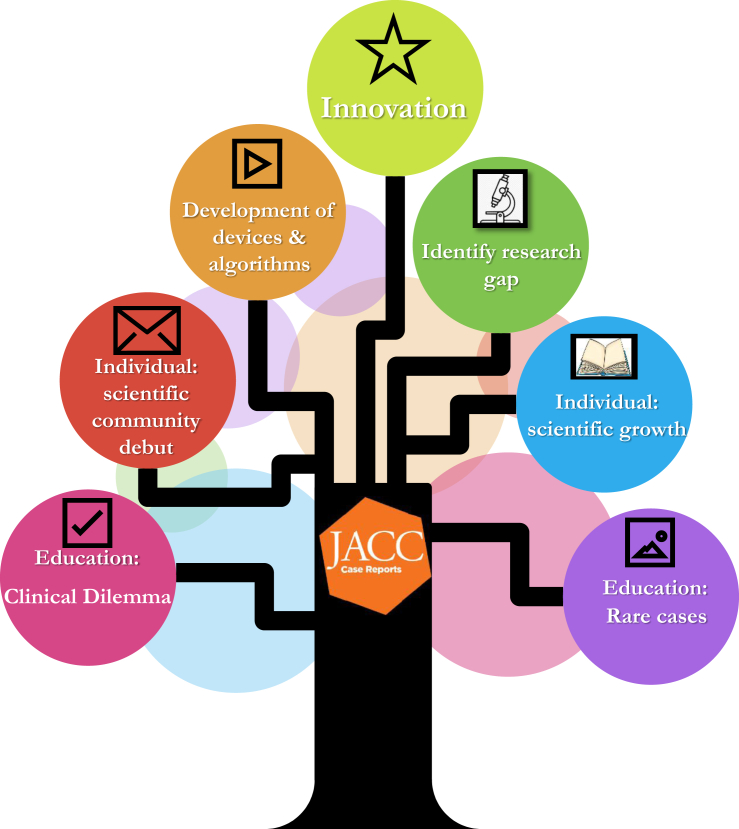

In the era of research, development, and innovation, are case reports still relevant? I asked myself that question when one of my trainees came to ask if he could write up one of our cases. The answer came effortlessly to me: YES! Ultimately, our role as physicians is to treat individual patients. Case reports offer insights from a single case that may highlight a novel procedural technique, rare condition, or innovative diagnostic tool, or simply provide a sensible algorithm for a clinical dilemma.

There are less known benefits to case reports. For example, well-written case reports can be a segue to larger research projects exploring the safety or feasibility of a certain technique. I remember reading about alternate access for transcatheter aortic valve replacement in a published case report. Soon after, the community generated valuable data on transcaval, transcarotid, and transaxillary approaches from registries such as the TVT (Transcatheter Valve Therapy) Registry or the French registry^1^ as well as some propensity-matched cohorts and even some prospective analyses.^1^, ^2^, ^3^, ^4^, ^5^ The field of medicine, and cardiology more specifically, is ever growing, and case reports can shed light on areas in which there is a paucity of data and little guidance to practitioners. They indicate areas in which research is necessary or in which refinement/development of new technologies is required.

Moreover, for the younger generations I would remind them that case reports can introduce them to the scientific community. As an Associate Editor for *JACC: Case Reports*, I recall reviewing a very well-written submission on spontaneous coronary artery dissection. The first author carefully selected and labeled the accompanying figures and very sensibly wrote the discussion points. I was impressed when I realized this was in fact a fellow in training, thinking what a debut! Guess what? I kept that fellow on my radar. I am not unique in that sense. Editorial Boards pay attention. Your work as authors or even reviewers matters and does not go unnoticed. In another instance, I recommended a fellow in training to review a manuscript that was assigned to me. Again, the commentary was spot-on and timely. Again: guess what? I asked the Editor-in-Chief to offer this reviewer an opportunity to write an accompanying editorial. Like case reports, editorials give the author a platform to showcase their prowess in critical appraisal and writing skills.

Although I have been emphasizing younger generations, I would venture to say case reports are for the rest of us, too. One of our early cases published in *JACC: Case Reports* was a patient with severe aortic stenosis and coarctation of the aorta.^6^ This elderly patient had advanced peripheral arterial disease with small caliber, tortuous, and calcified iliac arteries. We explored her subclavian artery as an alternative access. We still had concerns about caliber and calcification. This was our first venture into 3-dimentional printing, which permitted us to strategize and assess whether we would be able to deliver and implant a transcatheter heart valve safely by practicing using different access points on the printed model. This was a breakthrough for our center, particularly as our report was well received. We also discovered useful resources from the Da Vinci Anatomy Corner and Clinical Vignettes of *JACC: Case Reports* as we started our own 3-dimensional printing experience.^7^

Innovative initiatives, research theses, and progress can all start with a single case. It is important that we recognize that concept and teach the younger generations that the first step always matters, and case reports will always be relevant.
